# Chromium Stress Tolerance of a C4 (*Zea mays* L.) and C3 (*Vigna radiata* L.) Plants Primed with UV and Gamma-Treated *Bacillus subtilis*

**DOI:** 10.3390/microorganisms9112313

**Published:** 2021-11-08

**Authors:** Qasim Shahzad, Saqib Mahmood, Sadia Javed, Tariq Mushtaq

**Affiliations:** 1 Department of Biochemistry, Government College University, Faisalabad 38000, Pakistan; qasimkainth60@gmail.com; 2 Department of Botany, Government College University, Faisalabad 38000, Pakistan; drsaqibj@gmail.com; 3 Agrovision, Faisalabad 38000, Pakistan; tariq@agrovisions.com

**Keywords:** *Bacillus subtilis*, proline, chlorophyll, osmolyte, antioxidative enzymes, maize, mung bean

## Abstract

Chromium stress is one of the deleterious abiotic factors that reduce crop production. Two anatomically different crops (C3 and C4) were compared for their chromium (0 and 50 ppm) tolerance and responses towards *Bacillus subtilis* (*B. subtilis*). Strains of *B. subtilis* were exposed to UV (30–210 min) and gamma irradiation (1–4 KGy), and the best mutants were selected on petri plates containing selective markers. Maize and mungbean were supplied with selected strains or the parent strain in rooting medium, along with a nutrient broth. A completely randomized design (five replicates) was adopted using nutrient broth as a control. Stress negatively affected plants grown without strains. Mungbean was more sensitive towards stress and treatments, maize had better root and shoot fresh weights, root and shoot lengths, proline levels, and MDA and GR activity. All strains of *B. subtilis* (parent, γ-irradiated and UV-irradiated) enhanced proline, total soluble protein, chlorophyll a, a + b and a/b levels, with negligible effects upon antioxidant enzymes. Irradiated strains proved their superiority to the parent strain, with reductions in H_2_O_2_ and MDA content. With comparable benefits, γ and UV irradiation may be adopted in future based upon technical availability.

## 1. Introduction

Heavy metals are present in the vast majority of biotic and abiotic matter and frequently enter into the food chain. As a result of plant uptake, they negatively affect crop yield and food quality [1]. One of major reasons behind its spread is the rapid industrialization and use of contaminated waste water for irrigation without proper treatment [2]. Chromium is a heavy metal that negatively affects flora and fauna around the globe. It is found in a vast range of reservoirs including soil, ground water, and sediments. [3]. Plants growing under chromium stress uptake Cr (IV), which unavoidably changes plant morphology and physiology [4,5,6]. Absorbed Cr is not only located in roots but also translocates to the above-ground plant parts [7]. Therefore, it has opportunities to toxify whole-plant metabolism [8]. Chromium toxicity in plants causes reductions in growth and pigment content, as well as altered enzymatic activities [9,10,11].

**Figure 1:**
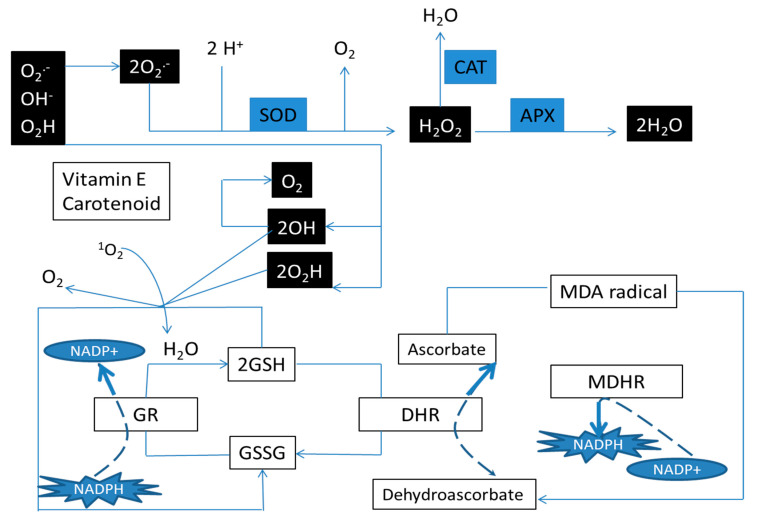
Enzymatic and non-enzymatic antioxidant system in plants. Enzymatic: Superoxide dismutase (SOD), catalase (CAT), ascorbate peroxidase (APX), Non-Enzymatic: oxidized glutathione (GSH), reduced (GSSG), monodehydroascorbate reductase (MDHR), dehydroascorbate reductase (DHR) and glutathione reductase (GR).

Stressed plants experience over-accumulation of reactive oxygen species (ROS) such as superoxide, peroxides, hydroxyl radicals and singlet oxygen. Excessive production of these species during photorespiration may destroy cell organelles. In addition, they can lead to cell death with enzymatic inhibition and oxidation of nucleic acids, lipids and proteins [12]. Plants produce enzymatic and non-enzymatic antioxidants to counteract ROS and resist/tolerate stressed conditions. Non-enzymatic antioxidants include ascorbic acid, β-carotene and tocopherols. Among the enzymatic antioxidants, catalase (CAT), peroxidase (POD), ascorbate peroxidase (APX) and superoxide dismutase (SOD) are the most commonly reported enzymes with antioxidant capacity (Figure 1). Another strategic solution that plants use for fighting against stresses is the accumulation of osmolytes such as soluble sugars, glycine, betaine, proline and proteins. These compatible osmolytes stabilize the cell molecular structure [13,14]. 

In case of longer exposure to stress or excessive contamination, natural neutralization of these ROS by antioxidant and osmolytes is not sufficient to alleviate the phytotoxicity of heavy metals in plants [15,16,17]. Plants need additional strategies to cope with such issues.

One possible economic and ecofriendly solution is the use of microbial communities, such as mycorrhizal fungi and plant growth-promoting bacteria (PGPB). Microbes known for their positive role in plant growth and development are being searched for their potential to support plants against abiotic and biotic stresses. To date, a number of microbes have been reported to promote the growth and development of normal and stressed plants; *Bacillus* is one of those [18,19,20,21]. 

Plants with differing structures and functions also vary in their adaptation to climatic conditions [22,23]. Based upon their anatomical differences, plants are generally categorized as C3/C4, where C3 plants have a distinctive leaf anatomy with a well-defined bundle sheath, chloroplast dimorphism, two carboxylation pathways in the bundle sheath cells and limited photorespiration. Generally, C3 plants are believed to have a temperate origin, while C4 plants have evolved in tropical and arid environments. While considering plant responses to their environmental conditions, anatomical and physiological distinctions may affect their adaption strategies [22].

After heavy metal uptake, in comparison to C4 plants, C3 species with higher biomass under elevated atmospheric carbon dioxide show a better potential for removing environmental contaminants [23]. Microbial association is reported to additionally favor tolerance strategies for elevated CO_2_. 

Maize (a C4 plant) and Mung bean (a C3 plant) are two important food crops. Maize is rich in essential amino acids, act as a gluten-free diet food, fortifies bones and boosts the nervous system. Mung bean (*Vigna radiata* L.) is an important small cash legume crop of Asia. With a good content of protein (21–24%), mung bean plants play and important role as food, fodder and green manure for soil improvement [24,25]. Better and enhanced yields of both crops may be improved by improving stress tolerance for better utilization of contaminated areas. Keeping in view the nutritive value of maize (C4) and mung bean (C3), chromium as a major constraint for plant growth and metabolism, and *B. subtilis* as a potential plant growth promoter, the present study was planned. Additionally, it was aimed to improve the *B. subtilis* strain by exposing it to UV and γ- irradiation. The major objective was to determine the more resistant/stress-tolerant strain and to determine the extent to which it can improve the growth and metabolism of a C4 (maize) and C3 (mung bean) species. 

## 2. Materials and Methods

### 2.1. Mutagenic Treatments and Selection of Mutants

The *Bacillus subtilis* (accession no. MW356848) strain used in this study was isolated by our research laboratory. This *B. subtilis* strain was inoculated in nutrient broth medium and exposed to UV (30, 60, 90, 120, 150, 180 and 210 min) at a single dose, lambda 360 for various time intervals and gamma radiations (1, 2, 3 and 4 kGy) at different dose rates for a fixed time interval. For UV mutation, the spores of *B. subtilis* (1 × 10^7^ spores/mL) were exposed to UV irradiation by using a UV lamp (Phillips, 20 W, λ360, distance of the lamp from the petri plates was 20 cm). The samples (1 mL) were withdrawn after 30 min until 240 min [26]. For Gamma mutation, four test tubes, each having 1 ml of spore suspension, were irradiated with γ-rays in a Cz137 source at different ranges of 1–4 KGy (1, 2, 3, 4). The irradiated spores after both treatments were diluted serially with nutrient broth medium and then an appropriate volume was spread on nutrient agar plates containing 0.2% triton X-100 as a colony restrictor. After overnight incubation at 37 °C, the colony-forming units were counted at a dilution giving rise to 100–200 colonies per plate, to determine the kill survival percentage. The irradiated spores were selected on agar plates containing 2-deoxy D-glucose (antimetabolite), used as a selective marker. The suppression/inhibition of the glycolysis process deprived the sensitive cells of ATP—the surviving strains, proving their resistance/stress tolerance, were counted on the plates and selected as the study mutants. 

### 2.2. Minimum Inhibitory Concentration 

The tolerance of each isolate was assessed for Cr stress with minimal inhibitory concentrations (MICs). For this purpose, different Cr concentrations (0 to 1500 mg/L potassium dichromate) were used. The highest Cr concentration at which no growth was observed on Cr-containing nutrient-agar media was considered as the MIC. 

### 2.3. Field Experiment

For field experiments, maize (Mallika) and mung bean (PRI-2018) seeds were obtained from Ayyub Agricultural Institute Faisalabad. Completely randomized experiments were designed with four replicates using plastic pots of 25 cm in depth and 12 cm in radius. Each pot was filled with 5 kg of clay-loam soil. In each pot, six seeds were sown. One week after sowing, the germinated seedlings were thinned to maintain five plants in each pot. The soil moisture content was maintained daily. Three weeks after germination, two chromium levels (0 ppm and 50 ppm) were maintained using potassium dichromate. After two weeks of chromium application, all the experimental units were subjected to root supplementations. Nutrient broth (13 g of oxoid nutrient broth powder (CM0001B) dissolved in 1 L of distilled water) was used to prepare the fungal supplements. Hence, plants exclusively supplemented with nutrient broth were considered as controls. This control was used as a comparison for the growth and biochemical attributes of maize and mung bean with three *B*. *subtilis* applications (parent, UV-irradiated and γ-irradiated strains in nutrient broth). After two weeks of application, harvests were taken to collect data on growth and biochemical attributes. Growth attributes included root and shoot length, and shoot fresh and dry weights. Biochemical attributes were determined as follows. 

### 2.4. Antioxidant Enzymes

Catalase activity was estimated following Cakmak & Horst [27]. For the determination of total protein, the extract was prepared by homogenizing 0.5 g of frozen leaf material in 0.05 M Tris-HCl buffer (pH 7). It was centrifuged in 4 °C for 30 min at 13,000 rpm/min. For catalase activity, 500 µL of 10 mM H_2_O_2_, sodium phosphate buffer (1400 µL of 25 mM) and 100 µL of crude enzyme extract were used. Decreases in absorbance were recorded at 240 nm for 1 min. Ascorbate oxidase activity was determined using the method of Wang et al. [28]. For peroxidase, H_2_O_2_ (500 µL of 5 mm), guaiacol (500 µL of 28 mM), and potassium phosphate buffer (1900 µL of 60 mM with pH 6.1) was used as the substrate and 100 µL crude extract was taken as the enzyme source. Differences in absorbance were recorded at 470 nm for 1 min with a spectrophotometer [29]. Leaf tissue (0.1 g) was ground in liquid nitrogen and then homogenized in 0.4 mL of phosphate buffer (50 mM at pH 7.0), ascorbic acid (1 mM), polyvinylpoly pyrrolidone (2%), triton (0.05%) and 1 mM ethylene diamine tetraacetic acid (EDTA). The mixture was centrifuged at 17,000× *g* for 20 min, and the supernatant containing 0.5 mM oxidized glutathione, 0.1 M Tris buffer (pH 7.8), 50 μM NADPH, 2 mM EDTA, and 20 μL of the extract was taken for further investigation. The assay was initiated by the addition of NADPH for 5 min at 25 °C. The oxidation reaction for measuring GR activity was followed by monitoring the absorbance at 340 nm using a UV-Vis spectrophotometer [30].

### 2.5. Lipid Peroxidation

For estimation of malondialdehyde (MDA), plant samples were homogenized in 3 mL of 0.1 trichloroacetic acid. The crude extract was mixed with the same volume of a 0.5% (*w*/*v*) tribarbitoric acid solution containing 20% (*w*/*v*) trichloroacetic acid. This was followed by 30 min heating and then rapid cooling. The absorbance of the supernatant was measured at 530 nm. Plant samples (frozen in liquid nitrogen and stored at 80 °C) were ground and 100 mg of the powder was homogenized with 5 mL of the solution containing 0.25 mL trichloroacetic acid (0.1%, 1 mL KI (1 M) and 0.5 mL potassium phosphate buffer (10 mM) The homogenate was centrifuged at 12,000× *g* for 15 min at 4 °C. The supernatant (500 μL) from each tube was kept at room temperature (about 25 °C) for 15 min [31].

### 2.6. Osmolyte and Protein Content

Fresh harvested leaf samples were frozen to 0 °C and then frozen to −40 °C. For sugar extraction, 0.1 g samples were chopped and added to 10 mL of 80% ethanol (*v*/*v*), followed by overnight shaking. Proline content was estimated by the method shown in [32]. Leaf samples (0.5 g) were homogenized in aqueous sulfosalicylic acid (3%), followed by centrifugation at 4 °C for 10 min at 10,000 rpm/min. The resultant supernatant of 1 mL, along with 1 mL acid ninhydrin and 1 mL of glacial acetic acid, was incubated at 100 °C for 1 h. The reaction mixture was extracted with 4 mL toluene on ice and the absorbance was noted at 520 nm. Soluble proteins were estimated from fresh leaves extracted with phosphate buffer solution (pH 7). From the filtrate, total protein was estimated using the method of Lowery et al. [33]. 

### 2.7. Photosynthetic Pigments

The chlorophyll content was determined following the method reported by Ptala [34]. For carotenoids, the estimation method of Davis [35] was adopted.

### 2.8. Statistical Analysis 

A two-way analysis of variance (ANOVA) of data for all attributes was carried out using CoStat a CoHort 6.4 for each crop independently. Mean values were compared using LSD.

## 3. Results 

A total of eleven bacterial strains—seven from UV and four from γ- irradiation treatment—were grown on petri plates containing nutrient agar medium along with 2-deoxy D-glucose as a selective marker, and only BSU-5, BSU-7 and BSγ-4 were found to be grown on petri plates (Table 1). All strains were also tested for their minimum inhibition concentration. Overall, BSU-7 and BSγ-4 was able to tolerate K_2_Cr_2_O_7_ up to 180 ppm in the culture media, whereas most of the remaining isolates were found to be tolerant to this salt up to 60 ppm, except for isolate BSU-5, which tolerated 90 ppm K_2_Cr_2_O_7_ (Table 2).

In the current study, out of eleven different strains, seven were exposed to UV irradiation (30–210) and four were exposed to γ-irradiation (1–4 kGy). The selection criteria for *B. subtilis* strains out of the seven UV and four γ- treatments was superiority in Cr-tolerance and growth on the selective marker. Among the isolated strains, two *B. subtilis* strains BSU-7 and BSγ-4 were selected (Table 1 and Table 2).

**Figure 2:**
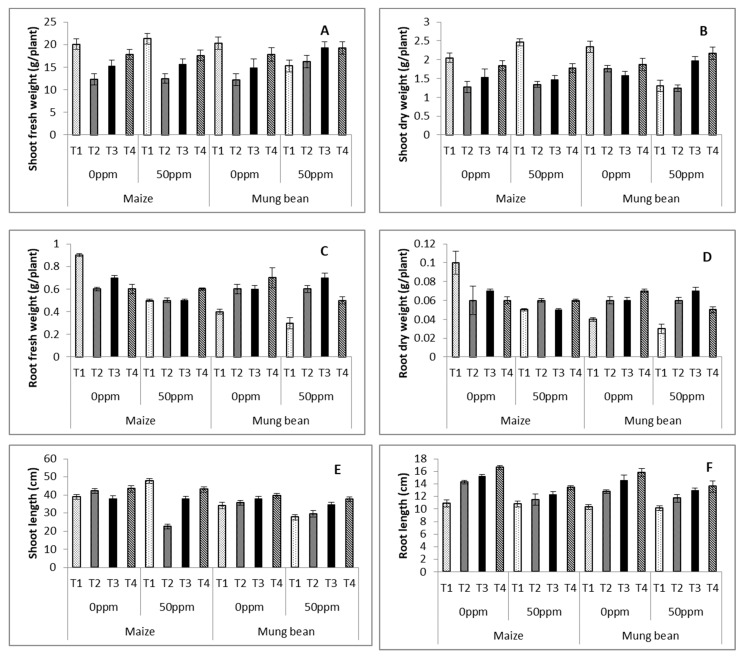
Shoot fresh (**A**) and dry (**B**) weights, root fresh (**C**) and dry (**D**) weights, shoot (**E**) and root (**F**) lengths, of maize (*Zea mays* L.) and mung bean (*Vigna radiata* L.) exogenously treated with *B. subtilis* under chromium stress conditions (mean ± S.E.). Here, T1 = Nutrient broth, T2 = Nutrient broth + *parent B. subtilis*, T3 = Nutrient broth + UV irradiated *B. subtilis* and T4 = Nutrient broth + γ-irradiated *B. subtilis*.

Data on the morphological attributes of maize and mung bean was subjected to analysis of variance. Results showed high significance (*p* ≤ 0.001) for stress, treatments and their interactions for shoot and root fresh and dry weight, and shoot and root lengths for maize and mung bean (Table 3 and Table 4; Figure 2A–D). Exogenously applied (UV and γ-irradiated) *B. subtilis* significantly improved shoot biomass exclusively in stressed mung bean, whereas for root weights (fresh and dry), with the exception of maize growing under 0 ppm stress, all plants experienced enhanced values of these attributes in nutrient medium supplemented with *B. subtilis,* as compared to the plants grown in nutrient medium without any strain. With regards to the lowering of root fresh and dry weights with stress (50 ppm), both crops experienced its reduction. Bacterial applications were more effective in mung bean, with marked improvement of these attributes. There was a marked suppression of shoot length in maize, but significant increase in the root length of both with the chromium stress (Figure 2E,F). *B. subtilis* improved the shoot as well as the root lengths of both stressed crops. The best lengths were displayed by the γ- irradiated plants, followed by the UV and Parent strains. 

Statistical analysis of enzymatic activities showed significance for the majority of parameters (*p* ≤ 0.001), along with significant interactions (Table 3 and Table 4). The activity of CAT was reduced in maize with chromium stress (50 ppm). Parent *B. subtilis* improved this attribute unanimously for both chromium levels in each crop. UV and γ –irradiated strains exclusively increased CAT activity in stressed maize. 

There was a negligible effect of stress upon SOD activity in control plants (nutrient broth without any strain). None of the strains were successful in improving its activity in comparison to the nutrient supplement without microbes (T1). 

The activity of POD was better displayed by mung bean in comparison to maize at both chromium levels. The response of *B. subtilis* strains was poorer than in the control (nutrient medium without strains), as with SOD.

APX enzymatic activity increased significantly (*p* ≤ 0.001) in mung bean with the increase of chromium levels (50 ppm). In maize, there was marked improvement in APX activity in plants supplemented with *B. subtilis* strains. In mung bean, only the parent *B. subtilis* has positive effects at 0 ppm chromium levels followed by γ and UV irradiated *B. subtilis*. 

**Figure 3:**
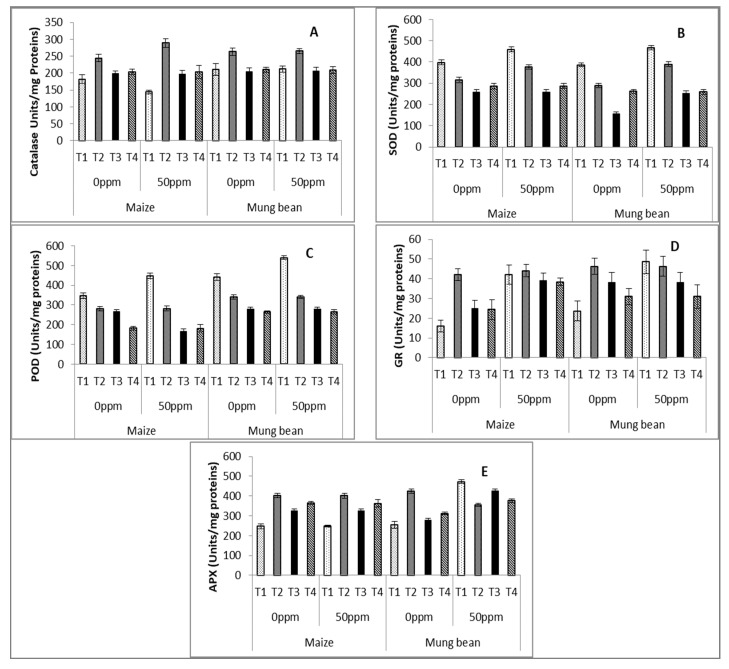
Estimation of activities of (**A**) superoxide dismutase (T1), (**B**) catalase (CAT), (**C**) peroxidase (POD), (**D**) glutathione reductase (GR) and (**E**) ascorbate oxidase (APX) enzymes of maize (*Zea mays* L.) and mung bean (*Vigna radiata* L.) exogenously treated with *Bacillus subtilis* under chromium stress conditions (mean ± S.E.). Here, T1 = Nutrient broth, T2 = Nutrient broth + *parent B. subtilis*, T3 = Nutrient broth + UV-irradiated *B. subtilis* and T4 = Nutrient broth + γ-irradiated *B. subtilis*.

GR activity significantly improved under stress, with a negligible effect of exogenous applications of strains to stressed plants. However, at 0 ppm conditions, plants supplemented with strains (T2, T3 & T4) showed higher GR activity compared to the plants solely supplied with nutrient medium (T1; Table 3 and Table 4; Figure 3A–E).

**Figure 4:**
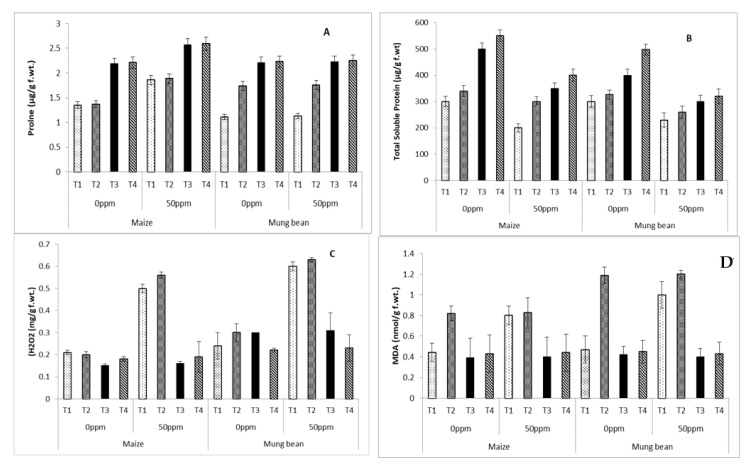
Biochemical attributes (**A**) Proline (**B**) Total soluble proteins (**C**) H_2_O_2_ (**D**) MDA of maize (*Zea mays* L.) and mung bean (*Vigna radiata* L.) exogenously treated with *B. subtilis* strains under chromium stress (0 ppm vs. 50 ppm) conditions (mean ± S.E.). Here, T1 = Nutrient broth, T2 = Nutrient broth *+ parent B. subtilis*, T3 = Nutrient broth + UV-irradiated *B. subtilis* and T4 = Nutrient broth + γ-irradiated *B. subtilis*.

Proline concentration increased significantly (*p* ≤ 0.05) in maize upon exposure to chromium, whereas mung bean exhibited similar patterns of proline accumulation at both chromium levels. Irradiated *B. subtilis* (UV and γ) improved this attribute in both crops. However, the wild *B. subtilis* was successful in increasing proline content only in mung bean (Table 3 and Table 4; Figure 4A). 

Total soluble protein was remarkably decreased under chromium stress conditions in plants lacking strains in rooting medium (T1; Table 3 and Table 4; Figure 4B), whereas *B subtilis* supplements (Parent, UV-irradiated and γ-irradiated) significantly enhanced the accumulation of total soluble proteins in both crops (Table 3 and Table 4; Figure 4B). The best total soluble protein levels were displayed by γ-irradiated strains followed by UV-irradiated and then parent *B subtilis* (Table 3 and Table 4; Figure 4B). Both crops showed marked enhancement of H_2_O_2_ with chromium stress in plants without any strains. Under controlled conditions, all supplemented (BS) and non-supplemented plants had similar levels of H_2_O_2_ (Table 3 and Table 4; Figure 4C). Under chromium stress (50 ppm), exogenously applied parent *B. subtilis* remained ineffective, although UV and γ-irradiated strains were successful in decreasing its levels in both crops. Furthermore, UV-irradiated mutants performed better in maize and γ-irradiated strains performed better in mung bean. An increase was observed in the malonidialdehyde (MDA) contents of both maize and mungbean with chromium stress in plants growing without strains (T1), and with parent strains (T2) in rooting medium. UV and γ-irradiated mutation of *B.subtilis* were equally effective in lowering MDA content (Table 3 and Table 4; Figure 4D).

**Figure 5:**
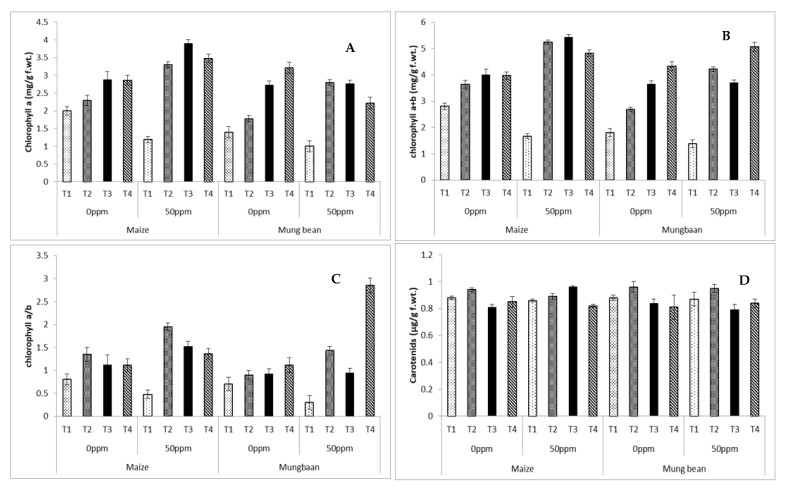
Photosynthetic pigments (**A**) chlorophyll a (**B**) chlorophyll a + b (**C**) chlorophyll a/b (**D**) carotenoids of maize (*Zea mays* L.) and mung bean (*Vigna radiata* L.) exogenously treated with different *B. subtilis* strains under chromium stress conditions (mean ± S.E.). Here T1 = Nutrient broth, T2 = Nutrient broth *+ parent B. subtilis*, T3 = Nutrient broth + UV-irradiated *B. subtilis* and T4 = Nutrient broth + γ-irradiated *B. subtilis*.

Chlorophyll a decreased significantly (*p* ≤ 0.001) under chromium stress in plants supplied only with nutrient broth in rooting medium. *B. subtilis* strains significantly (*p* ≤ 0.001) improved chlorophyll a content under various symbiotic conditions (Parent, UV and γ- irradiated) in maize and mung bean. Chlorophyll a + b experienced a marked decline with chromium application. All *B. subtilis* strains were successful in improving this attribute in both conditions of maize and mung bean. In both maize and mung bean, the ratio between chlorophyll a to b decreased with stress in plants grown with nutrient medium without any strain. All *B. subtilis* strains improved this ratio in both crops (Table 2 and Table 3; Figure 5). Results for carotenoid content were almost negligible with regards to comparisons of means. The only significant increase was noted in parent strain-treated plants in comparison to the nutrient medium-treated plants without *B. subtilis* strains (Figure 5D; Table 2 and Table 3).

## 4. Discussion

A substantial yield reduction in various economically important crops has been reported due to chromium stress [36]. Microbes like *B. subtilis* have received attention as a cheap source of bioremediation. Mutations of *B. subtilis* variably affected this potential of *B. subtilis* in the current project. In the present study, the best mutant strains were selected after UV and γ-irradiation treatments on the basis of their maximum potential to grow on a selective marker and their MIC values (Table 1 and Table 2). Chromium stress hindered plant growth in terms of fresh and dry biomass (Table 3 and Table 4; Figure 2), with significant inter-crop variation. Roots were directly exposed to chromium applied to the rooting medium; hence, both crops experienced a marked decline in biomass. Comparatively greater effects of chromium upon mung bean showed more sensitivity than maize, as it even experienced reduction of biomass in shoots. On the other hand, maize only experienced reductions in directly exposed organs (root). Maize maintained its shoot biomass under stress in plants without any strain supplement (Table 3 and Table 4; Figure 2A–D). Previously, Abou-Shanab et al. [19] and Weyens et al. [20] explored the bioremediation of heavy metal stress in plants. Our findings are in line with theirs, with the additional impact of gamma and UV mutations upon strains.

In addition to osmolytes, plants may fight against abiotic stresses with the help of antioxidants, including phenolics, carotenoids, catalase, SOD, POD, APX and GR [37,38,39]. CAT has the potential to reduce stress-induced ROS [37]. Decreased antioxidative activity under heavy metal stress has been reported formerly in maize [40] and other crops [35]. In the current project, there was a limited effect of strains upon these antioxidant enzymes. Exclusively, the parent strains improved CAT activity in both C4 and C3 species. For POD activity, the rooting medium supplemented with strains even showed reduced activity in comparison to the plants grown without strains. However, activity of POD was declined by *B. subtilis* strain application in plants growing under 0 ppm chromium. Our findings are in partial agreement with the literature [39]. 

Stressed plants generally attenuate oxidative stress with the help of antioxidant enzymes [41]. The currently used strains showed negligible potential for the improvement of antioxidant enzymes in both species used. 

Plants growing under heavy metal stress may also be able to cope using the synthesis/accumulation of some osmoprotectants [29,41,42,43]. These osmolytes help in the maintenance of cell turgor and osmo-regulation of stressed plants [42,43]. One of the most commonly reported osmoprotectants is leaf free proline. In a number of crops including sweet basil [44], rice [45] and many other crops [46], proline accumulation has been correlated with stress tolerance. In the present study, maize showed an enhanced accumulation of proline under chromium stress (0 ppm) in the absence of strains. This indicated better chromium tolerance in comparison to mung bean, which was also displayed in terms of growth attributes—particularly shoot and root weights (fresh and dry). These findings are in line with the reports of Anjum et al. [41]; they also observed better accumulation of osmolytes in maize plants growing with chromium stress. Strains of *B. subtilis* improved the proline content in both species, where mutants showing better accumulation of proline supported our hypothesis. Both species showed a similar reduction of total soluble proteins in plants without strains. Strain supplementation improved protein content, where the maximum was obtained by γ-irradiated *B. subtilis,* followed by UV and then parent strains. 

High oxidative stressors like chromium are believed to enhance H_2_O_2_ generation [47].

Shah et al., in their recent work [48], applied a *B. subtilis* strain to induce lead stress tolerance in *Solanum melongena*. There was a limited decline in H_2_O_2_ and MDA when the parent strain was applied alone, whereas enhanced reduction was noted when the strain was synergistically applied with silicon in stressed plants. In the current study, the responses of mutated strains were similar. However, the parent strains failed to decrease these stress indicators; the parent strains increased the levels of H_2_O_2_ and MDA. Greater H_2_O_2_ and MDA contents are indicative of higher lipid peroxidation. Hence, increases in these attributes in stressed plants grown with exclusive nutrient broth (T1) or with broth and parent strain (T1) combined supported the concept of irradiation for strain improvement (Table 3 and Table 4; Figure 4A,B). In contrast to our results, there are some reports where exogenously applied *Bacillus subtilis* has played a significant role in lowering H_2_O_2_ as well as MDA content [49] 

It is well known that chromium stress can considerably decrease photosynthetic pigment levels, which results in reduced plant growth and yield [45,46]. Chromium stress also leads to the reduction of the synthesis of green pigments (chlorophyll contents), resulting in decreased rates of photosynthesis [46]. In the present study, both crops exhibited a decline in chlorophyll a, chlorophyll a + b and chlorophyll a/b under stress in the absence of any *B. subtilis* strain (Figure 5C; Table 1). These findings are in agreement with Anjum et al. [41]. Reductions in chlorophyll may be due to the enhanced activities of chlorophyllase, involved in the breakdown of chlorophyll under stress conditions [49]. Several studies have reported the decrease of chlorophylls and carotenoids by chromium stress [7,42]. The chlorophyll a/b ratio is an important parameter indicative of plant metabolic efficiency; it is actually related to the phothosystem II (PSII) core and light-harvesting complex II (LHCII) ratio. PSII cores perceive excited energy from LHCII. Therefore, this chlorophyll a/b ratio indicates the activity of the plant photosynthetic machinery [50]. In the present study, *B. subtilis* strains proved themselves beneficial in the alleviation of chromium stress, with the improvement of photosynthetic pigments in addition to improved plant growth. Currently, the decline in the chlorophyll a/b ratio in chromium-stressed plants growing without strains and the treatment-based increases in this ratio supported our hypothesis. With minor variations, all strains improved photosynthetic activity (Figure 5). 

## 5. Conclusions

In the absence of *B. subtilis* strains, maize shows comparatively better chromium tolerance in comparison to mung bean (in terms of root and shoot fresh weight, and root and shoot lengths, proline levels, and MDA and GR activity). 

All strains of *B. subtilis* (parent, γ- irradiated and UV- irradiated) proved their positive role in the growth and metabolism of maize and mung bean grown under chromium stress—well shown by the enhancement of proline, total soluble protein, chlorophyll a, a + b and a/b levels. The irradiated strains (γ-and UV) further maintained their superiority over the parent strains, with better reductions in lipid peroxidation markers (H_2_O_2_ and MDA).

Overall, γ and UV-irradiated *B. subtilis* showed comparable results; therefore, either can be applied based upon technical availability. In future, similar strains may be applied to obtain metabolomics-level data for plants.

## Figures and Tables

**Table 1:** Selection of best mutants on the selective marker.

Strains	Growth on Selective Marker
BSU-1	-ve
BSU-2	-ve
BSU-3	-ve
BSU-4	+ve
BSU-5	-ve
BSU-6	-ve
BSU-7	+ve
BSγ-1	-ve
BSγ-2	-ve
BSγ-3	-ve
BSγ-4	+ve

+ve = growth was observed on Petri plates, -ve = no growth was observed on Petri plates.

**Table 2:** Minimum inhibitory concentration of isolates for Cr stress concentrations.

Strains	Cr Concentration (ppm)							
	0	30	60	90	120	150	180	210
BSU-1	+ve	+ve	-ve	-ve	-ve	-ve	-ve	-ve
BSU-2	+ve	+ve	+ve	-ve	-ve	-ve	-ve	-ve
BSU-3	+ve	+ve	-ve	-ve	-ve	-ve	-ve	-ve
BSU-4	+ve	+ve	+ve	-ve	-ve	-ve	-ve	-ve
BSU-5	+ve	+ve	-ve	+ve	-ve	-ve	-ve	-ve
BSU-6	+ve	+ve	-ve	-ve	-ve	-ve	-ve	-ve
BSU-7	+ve	+ve	+ve	+ve	+ve	+ve	+ve	-ve
BSγ-1	+ve	+ve	+ve	-ve	-ve	-ve	-ve	-ve
BSγ-2	+ve	+ve	-ve	-ve	-ve	-ve	-ve	-ve
BSγ-3	+ve	+ve	-ve	-ve	-ve	-ve	-ve	-ve
BSγ-4	+ve	+ve	+ve	+ve	+ve	+ve	+ve	-ve

+ve = growth was observed on Petri plates, -ve = no growth was observed on Petri plates.

**Table 3:** Mean Squares from two way analysis of variance data for different morphological and biochemical indicators of Maize (*Zea mays* L.) treated with *Bacillus subtilis* under chromium stress.

Source of Variation	df	Shoot FW	Shoot DW	Root FW	Root DW	Shoot Length
Stress (S)	2	0.9325 ***	0.1386 ***	0.0902 ***	0.0856 ***	0.680 ***
Treatment (T)	3	113.212 ***	1.282 ***	29.10 ***	1.064 ***	270.12 ***
S × T	6	0.346 ***	0.720 ***	0.234 ***	0.0119 ***	0.3113 ***
		**Root Length**	**Proline**	**MDA**	**H_2_O_2_**	**Protein**
Stress (S)	2	0.285 ***	0.0077 ***	3.083 ***	0.0013 ***	2.586 ***
Treatment (T)	3	1150.88 ***	0.499 ***	0.3531 ***	0.3165 ***	0.105 ***
S × T	6	0.547 ***	1.409 ^ns^	0.0010 ***	1.57 ^ns^	3.861 ^ns^
		**Catalase**	**APX**	**GR**	**POD**	**SOD**
Stress (S)	2	8.694 ^ns^	2.194 ***	0.0017 *	8.0833 ***	11.083 ***
Treatment (T)	3	193,581.7 ***	67,589.06 ***	1078.4 ***	202,487.74 ***	98,622.4 ***
S × T	6	15.95 ^ns^	1.3425 ***	3.416 ^ns^	0.0555 ***	0.0833 ^ns^
		**Chl a**	**Chl a + b**	**Car**	**Chla/b**	
Stress (S)	2	0.0010 ^ns^	2.612 ***	0.0016 ***	2.61 ***	
Treatment (T)	3	68.135 ***	7.59 ***	0.0120 ***	7.62 ***	
S × T	6	0.0029 ^ns^	2.41 ***	0.0084 ***	2.391 ***	

ns = non-significant; * and *** = significant at 0.05, 0.01 and 0.001 levels, respectively.

**Table 4:** Mean Squares from two way analysis of variance data for different morphological and biochemical indicators of Mung bean (*Vigna radiata* L.) treated with *Bacillus subtilis* under chromium stress.

Source of Variation	df	Shoot FW	Shoot DW	Root FW	Root DW	Shoot Length
Stress (S)	2	9.284 ***	1.425 ***	0.0033 ***	0.0350 ***	0.0099 ***
Treatment (T)	3	0.0096 ***	2.469 ***	0.0029 ***	1.0230 ***	253.78 ***
S × T	6	0.0011 ***	3.491 ***	0.0012 ***	0.0092 ***	0.1922 ***
		**Root Length**	**Proline**	**MDA**	**H_2_O_2_**	**Protein**
Stress (S)	2	0.4967 ***	0.0052 ***	2.860 ***	0.0015 ***	5.361 ***
Treatment (T)	3	133.16 ***	1.1732 ***	1.281 ***	0.313 ***	0.141 ***
S × T	6	0.2990 ***	1.111 ^ns^	3.675 ***	1.38 ^ns^	1.484 ^ns^
		**Catalase**	**APX**	**GR**	**POD**	**SOD**
Stress (S)	2	6.583 ***	3166.5 ***	7.583 ***	6.0277 ***	6.194 ***
Treatment (T)	3	284,498.0 ***	8134.02 ***	1411.4 ***	178,010.19 ***	178,701.3 ***
S × T	6	0.879 ***	25,551.1 ***	2.5 ^ns^	9.435 ***	2.083 ***
		**Chl a**	**Chl a + b**	**Car**	**Chl a/b**	
Stress (S)	2	0.0015 ^ns^	2.6 ***	6.194 **	2.61 ***	
Treatment (T)	3	60.102 ***	7.63 ***	0.0350 ***	7.62 ***	
S × T	6	2.0833 ^ns^	2.4 ***	0.0025 ***	2.391 ***	

ns = non-significant; ** and *** = significant at 0.01 and 0.001 levels, respectively.

## Sample Availability

All strains, plant materials and chemicals are available from the authors.
